# Correction: Precise measurement of selenium isotopes by HG-MC-ICPMS using a 76–78 double-spike

**DOI:** 10.1039/d2ja90030k

**Published:** 2022-06-16

**Authors:** Marie-Laure Pons, Marc-Alban Millet, Geoff N. Nowell, Sambuddha Misra, Helen M. Williams

**Affiliations:** The University of Cambridge, Department of Earth Sciences Downing St Cambridge CB2 3EQ UK dr.marie.laure.pons@gmail.com; CNRS, Aix Marseille Univ, IRD, INRA, Coll France, CEREGE 13545 Aix en Provence France; Cardiff University, School of Earth and Ocean Sciences Main Building, Park Pl Cardiff CF10 3AT UK; Durham University, Department of Earth Sciences Elvet Hill Durham DH1 3LE UK; Indian Institute of Science, Centre for Earth Sciences Bengaluru India

## Abstract

Correction for ‘Precise measurement of selenium isotopes by HG-MC-ICPMS using a 76–78 double-spike’ by Marie-Laure Pons *et al.*, *J. Anal. At. Spectrom.*, 2020, **35**, 320–330, https://doi.org/10.1030/c9ja00331b.

The authors regret an error in Fig. 1. The correct figure is as follows:

**Fig. 1:**
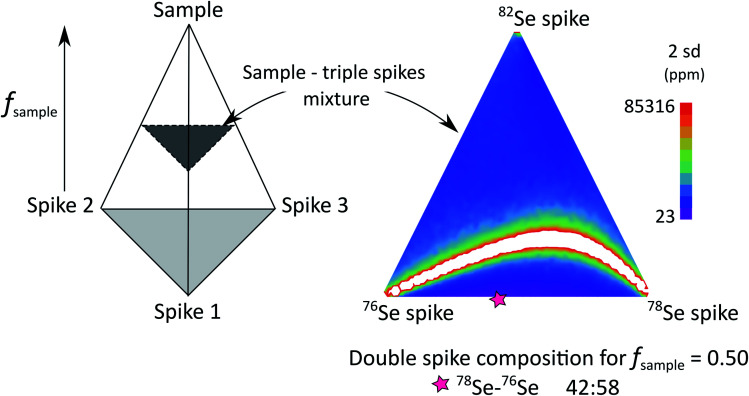
Schematic representation of all the possible spike–sample mixtures investigated in our triple spike Monte-Carlo simulation. All compositions are enclosed in a tetrahedron where the top apex is the standard composition (*i.e.*, natural stable isotope composition) and base apexes (light grey area) are the individual spikes. In this tetrahedron, sections parallel to the base (represented in dark grey) contain all possible triple spike mixtures mixed with the same amount of natural sample.

The authors also regret an error in the graphical abstract image. The correct graphical abstract image is as follows:
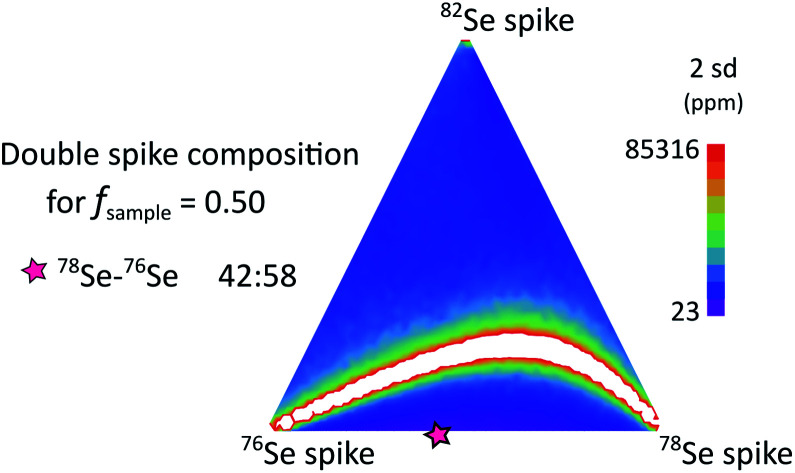


The Royal Society of Chemistry apologises for these errors and any consequent inconvenience to authors and readers.

## Supplementary Material

